# HPIDB - a unified resource for host-pathogen interactions

**DOI:** 10.1186/1471-2105-11-S6-S16

**Published:** 2010-10-07

**Authors:** Ranjit Kumar, Bindu Nanduri

**Affiliations:** 1 College of Veterinary Medicine, Mississippi State University, Mississippi State, MS 39762, USA; 2 Institute for Digital Biology, Mississippi State University, Mississippi State, MS 39762, USA

## Abstract

**Background:**

Protein-protein interactions (PPIs) play a crucial role in initiating infection in a host-pathogen system. Identification of these PPIs is important for understanding the underlying biological mechanism of infection and identifying putative drug targets. Database resources for studying host-pathogen systems are scarce and are either host specific or dedicated to specific pathogens.

**Results:**

Here we describe "HPIDB” a host-pathogen PPI database, which will serve as a unified resource for host-pathogen interactions. Specifically, HPIDB integrates experimental PPIs from several public databases into a single, non-redundant web accessible resource. The database can be searched with a variety of options such as sequence identifiers, symbol, taxonomy, publication, author, or interaction type. The output is provided in a tab delimited text file format that is compatible with Cytoscape, an open source resource for PPI visualization. HPIDB allows the user to search protein sequences using BLASTP to retrieve homologous host/pathogen sequences. For high-throughput analysis, the user can search multiple protein sequences at a time using BLASTP and obtain results in tabular and sequence alignment formats. The taxonomic categorization of proteins (bacterial, viral, fungi, etc.) involved in PPI enables the user to perform category specific BLASTP searches. In addition, a new tool is introduced, which allows searching for homologous host-pathogen interactions in the HPIDB database.

**Conclusions:**

HPIDB is a unified, comprehensive resource for host-pathogen PPIs. The user interface provides new features and tools helpful for studying host-pathogen interactions. HPIDB can be accessed at http://agbase.msstate.edu/hpi/main.html.

## Background

Proteins are the work horses of living organisms; they interact with other proteins to carry out most of the biological functions such as signal transduction, protein transport, immune response and other essential functions. PPIs can be classified into two main categories: "Intra-species PPI," where two proteins from the same species interact with each other, and "Inter-species PPI," where two proteins from two different species interact. Host-pathogen protein–protein interactions (HPIs) that play a vital role in initiating infection are a subset of inter-species interactions. Identification and study of HPIs is critical for understanding molecular mechanisms of infection and subsequent development of drug targets.

Although a number of databases that store PPIs are described in the literature [1-3], only a few databases contain inter-species interactions [4-7]. Thus resources for studying host-pathogen interactions are very limited and users have to access multiple databases followed by manual curation to get the desired set of HPIs. Although there are few efforts toward developing dedicated host-pathogen interaction databases but the existing resources are limited in scope or confined to a limited number of species. The PIG (pathogen Interaction gateway) database provides a collection of HPIs from different resources, but is limited to only one host species, i.e. “human" [8]. Also, the search options in the PIG database are limited to gene identifiers, and BLASTP alignment results are not displayed for sequence searches, so the user cannot evaluate the quality of the alignment. Furthermore, BLASTP cannot be performed in batch mode (multiple sequences at a time), making it difficult to apply for modeling high throughput datasets. Another database "Phi-base" catalogues information about experimentally verified pathogenicity, virulence and effector genes from fungal, oomycete and bacterial pathogens, but does not provide any PPI information [9]. VirhostNet database is dedicated for only virus related PPIs [10]. Other pathogen specific databases have also been reported [11]. Apart from limited availability of experimental HPIs, very few computational approaches have been reported for predicting HPIs. Protein domain profiles of existing intra-species PPIs were used to predict the interaction between human and plasmodium proteins [12]. In another study, existing intra-species PPIs were used to identify orthologous interactions (interologs), which were then used to predict inter-species interactions [13,14]. Both of these computational studies use intra-species PPIs to predict inter-species interactions. Furthermore, they do not provide any web based tool for predicting HPI. In another approach, experimentally identified PPIs are used to search for homologous PPIs to transfer annotations to a new species [15], but the provided tool is limited to predicting intra-species interactions, and has not been applied to predict HPIs.

Here we describe HPIDB, a unified resource that integrates HPIs from multiple resources into a single, non-redundant set in a user friendly web accessible format. The user interface provides multiple options for querying the database content and facilitates BLASTP [16] based sequence searches. It also provides a web based tool which searches for existing homologous HPIs in the HPIDB, which can be used to transfer HPIs to other species.

## Construction and content

**Figure 1:**
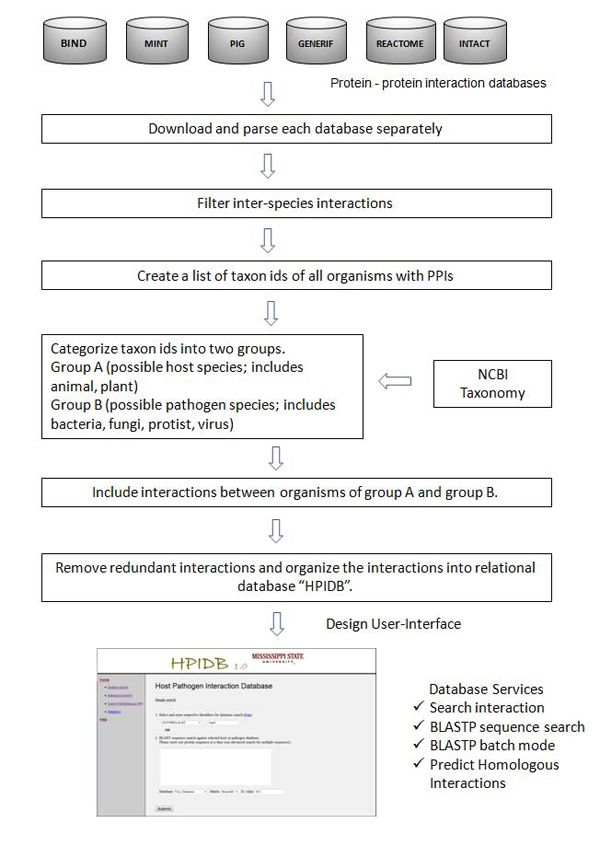
**Workflow for the construction of HPIDB.** This diagram illustrates data retrieval from various databases, parsing, storage and usage. It also includes a web based user interface which interacts with database.

The HPIDB database is implemented using MYSQL 5.1, with the user interface and web server designed using CGI and Perl. Figure 1 illustrates the workflow of HPIDB and shows data retrieval from various public resources, parsing, storage and specific usage. PPIs from various resources were collected into a single repository. Individual scripts were written to download and parse the data from each PPI database into one unified format. The database schema is available on the website (http://agbase.msstate.edu/hpi/main.html). Only inter-species interactions were selected from the repository. For proper classification of PPI into HPI, we classified the taxon ids present in the inter-species interactions into two groups: group A had the taxonomic ids for host species (includes human, plant, animals, etc.) and group B had the taxonomic ids of pathogenic species (includes bacteria, virus, fungi, protists, etc.). All PPIs where a protein from “group A” interacts with a protein from “group B” are selected and considered as possible Host pathogen interactions (HPIs). To eliminate the possibility of redundant HPIs, all entries were converted into UniProt accession and duplicate entries were eliminated. Where UniProt accession conversion was not successful, duplicate PPI entries were removed based on the protein sequence identity. All the identified interactions were organized into a relational database with additional features like synonym, taxon id, sequence, function, interaction type, experimental information used to identify PPI, and literature information (PubMed id and author information). A web based user interface was designed to query the database using various identifiers, perform BLASTP based protein sequence searches and provide a tool for searching homologous interactions.

All the protein sequences in the database are grouped into major taxonomic groups like plant, animal, bacteria, virus, fungi, and protist. BLASTP sequence alignment functionality was added to the database to search against similar protein sequences. Scripts were written to perform the BLASTP sequence searches in batch mode (search multiple protein sequences at a time) and process the results. The taxonomic classification of protein sequences is integrated with BLASTP, to search only a particular group of sequence databases such as bacteria, virus, animal, all pathogen, all host, etc. Taking advantage of this taxonomic classification of proteins into host and pathogen, we designed a "Search Homologous HPIs" module within the HPIDB. This tool enables the user to search for homologous HPIs in the database for a given set of host and pathogen protein sequences. Internally, this tool is executed in three steps:

1. Input user provided host protein sequences (A) in FASTA format, conduct BLASTP searches against all host proteins in HPIDB and output homologous host protein (HA). 

2. Input user provided pathogen protein sequences (B) in FASTA format, conduct BLASTP searches against all pathogen protein in HPIDB and output homologous pathogen protein (HB).

3. Combine the results from step 1 and step 2; any interactions found between HA and HB in HPIDB database are called homologous host-pathogen interactions for proteins A-B. This module provides the user with a set of homologous interactions for further analysis and wherever possible, the results obtained in this module (for example, HA-HB) can be used to transfer annotation to a new species (A-B).

Currently HPIDB contains 22,841 interactions between 49 host and 319 pathogen species. Table 1 shows the prominent set of host and pathogen species represented in HPIDB.

**Table 1:** Summary of representative host and pathogen species in HPIDB.

Summary of Host Pathogen PPI stored in HPIDB		
**Taxon id**	**Name**	**Number of PPI**

**Host**		
9606	*Homo sapiens*	22386
10090	*Mus musculus*	147
3702	*Arabidopsis thaliana*	99
10116	*Rattus norvegicus*	53
9913	*Bos taurus*	30
9031	*Gallus gallus*	19
		
**Pathogen**		
1392	*Bacillus anthracis*	6965
11676	Human immunodeficiency virus 1	3723
119856	*Francisella tularensis subsp. tularensis*	1341
10376	Human herpesvirus 4	354
11685	Human immunodeficiency virus type 1 (ARV2/SF2 ISOLATE)	344
11696	HIV-1 M:B_MN	341
11689	Human immunodeficiency virus type 1 (ELI ISOLATE)	340
362651	Human immunodeficiency virus type 1 (YU-2 isolate)	340
11688	Human immunodeficiency virus type 1 (JRCSF ISOLATE)	338
11697	Human immunodeficiency virus type 1 (MAL ISOLATE)	338
11701	Human immunodeficiency virus type 1 (RF/HAT ISOLATE)	338
4932	*Saccharomyces cerevisiae*	337
11678	Human immunodeficiency virus type 1 BH10	320
211044	Influenza A virus (A/Puerto Rico/8/34(H1N1))	303
11683	Human immunodeficiency virus type 1 (Z2/CDC-Z34 ISOLATE)	296
11699	Human immunodeficiency virus type 1 (OYI ISOLATE)	294
11686	Human immunodeficiency virus type 1 (BRU ISOLATE)	292
333284	Hepatitis C virus (isolate Con1)	283

## Utility and discussion

The database can be accessed using the web interface, which is divided into three separate modules based on specific user needs. Alternatively the whole database can be downloaded from the website in tab delimited file format.

### Using the web interface

The web interface is divided into three separate modules:

1. "Simple search" is used to search the database based on user defined identifiers like UniProt id, alias name, symbol, taxonomy id, interaction type, literature information (like PubMed id, author, etc.). The results include a set of host-pathogen PPIs along with additional taxonomic categorization in tabular format. A search result with all information about all PPIs is available for download in tab delimited text format, which can be further used in other programs like Cytoscape [17] for network construction and visualization. Protein information is also hyperlinked to other databases for access to available functional annotation, Gene Ontology, and PubMed references. The BLASTP interface is provided, which can be used to determine if a similar protein is involved in the HPI. The user can adjust the BLASTP search parameters and database category (otherwise, default values are provided). The BLASTP search results are returned in both tabular format (for quick analysis) and standard output format (with pair-wise alignment) for user convenience. The results in tabular format are further referenced back to the entries in original database.

2. "Advanced BLAST search" provides the ability to perform BLASTP sequence searches in batch mode. Users can provide more than one protein sequence at a time in FASTA format. Apart from the features provided in a simple BLAST query, users have the option to either get the top hit result for each query or get multiple hits below a user specified E-value.

3. "Search Homologous HPIs" is used to search for homologous HPIs in the HPIDB. For a given set of host and pathogen proteins, first the program tries to identify similar host and pathogenic proteins (based on BLASTP results) in the database. If the identified homologs were involved in HPI interaction in HPIDB, it would be called a homologous HPI. This tool can also be used for only host or pathogen sequences to search homologous host/pathogen proteins and their interacting partners. 

**Figure 2:**
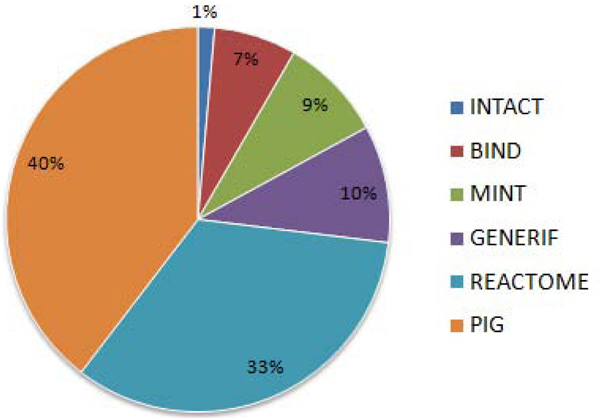
Distribution of PPIs from various databases present in HPIDB.

The user interface includes a statistics page which summarizes the interactions present in the database (Figure 2). A help file is included, which explains the database schema and the workflow for using the tools with the sample input and output files. More databases can be easily added to HPIDB and it will be updated every three months. In the future, we plan to extend the homologous HPI prediction and combine it with the protein domain profiles from the HPIDB proteins to develop a computational HPI prediction tool.

Here we describe three case studies that demonstrate the utility of HPIDB to researchers in achieving their objective:

### Case study 1

A researcher is studying a particular bacterial species and its related strains that cause infection in humans and animals. In order to identify the host specificity as well as the source for varying infectivity of the bacterial strains of interest, he wants to get a list of all host-pathogen PPIs available for each strain. 

Instead of searching various databases and filtering inter-species PPIs from them individually, this researcher can search the HPIDB using the "simple search" feature and use the taxon ids of all the bacterial species in the search field (one by one) to get the desired PPI dataset.

### Case study 2

**A researcher has sequenced a new bacterial genome and wants to identify proteins in the genome that are similar to known bacterial proteins involved in host-pathogen interactions.** Existing PPI/HPI resources do not provide sequence searches for a particular taxonomic category. An advantage that HPIDB has over other comparable databases is that it provides the categorization of pathogen protein sequences into categories like bacteria, fungi, protist, virus, etc. and host protein sequences into categories such as animal, plant, etc. based on taxonomy. The researcher can use the "Advanced BLAST search" option to perform a BLASTP search in batch mode with all protein sequences from the genome against the bacterial protein database. 

### Case study 3

**A user is studying pneumonia, a disease caused by the human pathogen *Streptococcus  pneumoniae*. In the absence of any experimental PPIs between human and *S.pneumoniae,* the user needs to identify putative HPIs based on similar homologous interactions (BLASTP E-value < 10^-20^) present in the database to generate a testable hypothesis.** Currently, there is no web based tool available that enables the user to search for homologous HPIs. *S*. *pneumoniae* proteins sequences (2105) were downloaded from NCBI (ftp://ftp.ncbi.nih.gov/genomes/Bacteria/Streptococcus_pneumoniae_TIGR4/NC_003028.faa). In the HPIDB, the “Search Homologous HPIs" can be selected to identify homologous HPIs. Here option A is selected as HPIDB already has human proteins in the database and no further predictions for homologs host proteins are desired. “Form A” should be used which inputs the pathogen protein sequences in FASTA format, the BLASTP parameters can be set to have an E-value < 10^-20^ and the “bacterial proteins” should to selected as database. When we conducted this search, we identified 2001 HPIs between 492 pathogen proteins and 1153 host proteins (mostly human). The dataset can be used further to transfer the homologous interactions and to predict new interactions between human and *S.pneumoniae.* For example, the predicted interactions include previously known virulence factors of *S*. *pneumoniae *[18] like 3 different capsule proteins (SP0350, SP0357, SP0360), trigger factor (SP0400), exoenzyme enolase (SP1128), pneumolysin (SP1923), Streptococcal lipoprotein rotamase (2012) and serine protease (SP2239) (Additional File 1). Using the output from HPIDB in Cytoscape, one can start exploring the interaction network of all virulence proteins mentioned above with human proteins (Additional File 2).

## Conclusions

We developed a new host-pathogen protein-protein interaction database "HPIDB" which will serve as a unified and comprehensive resource for HPIs. The user interface provides multiple options to search the database. HPIDB allows high throughput sequence searches in which the user can submit multiple protein sequences at a time and search against a selected taxonomic category. HPIDB also includes a tool that can search for homologous HPIs in the database for user provided sequences. All these features of HPIDB will be helpful for studying host-pathogen interactions. 

## Availability and requirements

Project name: HPIDB

Project home page: http://agbase.msstate.edu/hpi/main.html

Restrictions for use by non-academics: none

## List of abbreviations used

PPI: (protein-protein interaction); PPIs: (protein-protein interactions) ; HPI: (host-pathogen interaction) ; HPIs: (host-pathogen interactions); HPIDB: (host-pathogen interaction database)

## Authors' contributions

RK contributed to the design of HPIDB, wrote all of the scripts for the database construction and implementation, and wrote the draft of this manuscript. BN conceived this study, contributed to the design of HPIDB, helped to analyze and interpret the results, and helped to draft the manuscript. All authors read and approved the final manuscript.

## Competing interests

All authors have declared that there are no competing interests.

## Supplementary Material

Additional File 1 Description: Selected homologous HPIs identified during Case study 3 for *S. pneumoniae* and human proteins. Click here for file

Additional File 2 Description: HPI network for selected *S*. *pneumoniae* and human proteins visualized using Cytoscape. Click here for file
