# Supplementary figures and images for: HPIDB - a unified resource for host-pathogen interactions
Source: BMC Bioinformatics. 2010 Oct 7;11(Suppl 6):S16. doi: 10.1186/1471-2105-11-S6-S16 (PMC3026363; doi:10.1186/1471-2105-11-S6-S16)

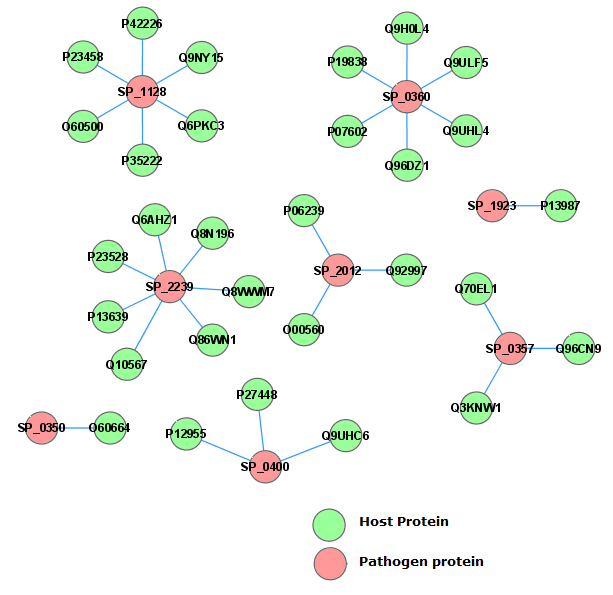

Supplement: Additional File 2 — Description: HPI network for selected S. pneumoniae and human proteins visualized using Cytoscape. [file 1471-2105-11-S6-S16-S2.tif]
